# Functional Analysis of an Inducible Promoter Driven by Activation Signals from a Chimeric Antigen Receptor

**DOI:** 10.1016/j.omto.2018.11.003

**Published:** 2018-12-01

**Authors:** Ryosuke Uchibori, Takeshi Teruya, Hiroyuki Ido, Ken Ohmine, Yoshihide Sehara, Masashi Urabe, Hiroaki Mizukami, Junichi Mineno, Keiya Ozawa

**Affiliations:** 1 Division of Immuno-Gene and Cell Therapy, Jichi Medical University, Shimotsuke, Japan; 2 Division of Genetic Therapeutics, Center for Molecular Medicine, Jichi Medical University, Shimotsuke, Japan; 3 CDM Center, Takara Bio Inc., Kusatsu, Japan; 4 Division of Hematology, Department of Medicine, Jichi Medical University, Shimotsuke, Japan

**Keywords:** inducible gene expression, chimeric antigen receptor, adoptive T cell therapy, B cell lymphoma, CD19, tumor targeting, *in vivo* imaging

## Abstract

Adoptive transfer of T cells expressing a chimeric antigen receptor (CAR) is a promising cell-based anticancer therapy. Although clinical studies of this approach show therapeutic efficacy, additional genetic modification is necessary to enhance the efficacy and safety of CAR-T cells. For example, production of an antitumor cytokine from CAR-T cells can potentially enhance their tumor-killing activity, but there are concerns that constitutive expression of anticancer molecules will cause systemic side effects. Therefore, it is important that exogenous gene expression is confined to the tumor locality. Here, we aimed to develop an inducible promoter driven by activation signals from a CAR. Transgene expression in T cells transduced with the CD19-targeted CAR and an inducible promoter, including inducible reporter genes (CAR-T/iReporter), was only induced strongly by co-culture with CD19-positive target cells. CAR-T/iReporter cells also showed redirected cytolysis toward CD19-positive, but not CD19-negative, tumor cells. Overall, our study indicated that the inducible promoter was selectively driven by activation signals from the CAR, and transduction with the inducible promoter did not affect original effector activities including interleukin-2 and interferon-γ production and the antitumor activity of CAR-redirected cytotoxic T lymphocytes. Moreover, this inducible promoter permits visualization and quantification of the activation status in CAR-T cells.

## Introduction

Adoptive transfer of T cells expressing a chimeric antigen receptor (CAR) is a promising cell-based anticancer therapy.^1^, ^2^, ^3^, ^4^, ^5^ This approach involves both cellular and humoral immune responses by assembly of an antigen-binding moiety, most commonly a single chain variable fragment (scFv) derived from a monoclonal antibody, together with an activating immune receptor, such as the intracellular domain from CD3ζ and/or CD28. Once the CAR is expressed at the surface of modified T cells and upon binding of the scFv to its antigen, an activation signal is transmitted into the T cell, which in turn triggers its effector functions against the target cell.^6^, ^7^, ^8^ As a result, T cells are activated and can efficiently eliminate tumor cells by secretion of interferon (IFN)-γ, perforin, and granzymes as well as the expression of Fas ligand (FasL) and tumor necrosis factor (TNF)-related apoptosis inducing ligand (TRAIL).^6^, ^9^, ^10^ In addition, the secretion of various cytokines, such as interleukin (IL)-2 and TNF-α, activates other tumor-infiltrating immune cells.^10^, ^11^ Although clinical studies of this approach show therapeutic efficacy, additional genetic modification is necessary for enhancement of the therapeutic efficacy and safety of CAR-T cells.

TCR and CAR activations promote the calcium-signaling pathway.^12^, ^13^ Generally, CARs containing the CD3ζ and/or CD28 signaling domain have been used to show therapeutic efficacy.^6^, ^7^, ^10^ An early event in such CAR activation is phosphorylation of immunoreceptor tyrosine-based activation motifs on the cytosolic side of CD3ζ by lymphocyte protein tyrosine kinase (Lck).^14^, ^15^, ^16^, ^17^, ^18^, ^19^ Then, ζ-chain-associated protein kinase (Zap-70) is recruited to the CAR, where it becomes activated. Inositol trisphosphate (IP3) triggers the entry of extracellular Ca^2+^ into cells. Calcium-bound calmodulin (Ca^2+^/CaM) activates the phosphatase calcineurin, which promotes transcription of genes regulated by nuclear factor of activated T cells (NFAT), including IL-2.^18^, ^19^, ^20^

Therefore, an NFAT-dependent luciferase reporter system can be used to monitor the activity of calcineurin-NFAT signaling that indicates the activation status of T cells.^21^

Although combination with an inducible promoter including IL-12 or IL-18 production in CAR or TCR therapy has been described in a previous study and even in clinical trials,^22^, ^23^, ^24^, ^25^, ^26^, ^27^ detailed functions of the inducible promoter have not been analyzed.

Here, we show the potential of this inducible expression system to visualize and quantify the activation status of CAR-expressing T cells.

## Results

### Development of Inducible Promoters Using Jurkat Cells That Constitutively Express a CD19-CAR

**Figure 1:**
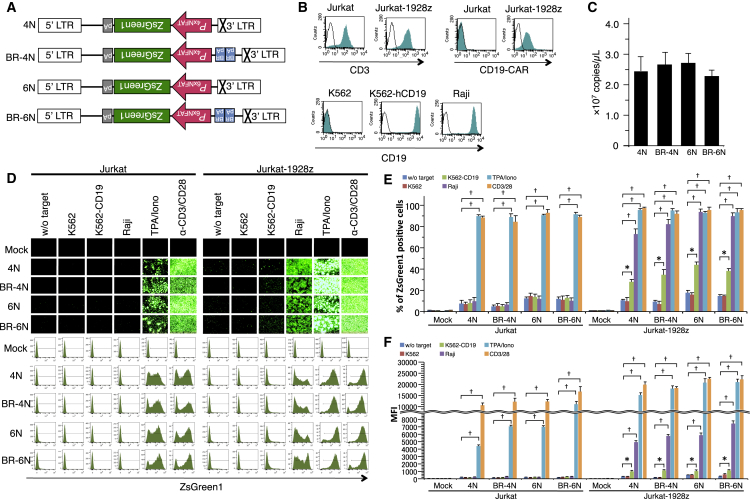
ZsGreen1 Expression Driven by Nuclear Factor of Activated T Cell-Responsive Elements in Jurkat Cells (A) Schematic representation of retroviral vectors: 4N, 6N, BR-4N, and BR-6N; LTR, long-terminal repeat; NFAT, composite NFAT-responsive promoter element; pA, polyadenylation signal; BR, background reduction signal. (B) Jurkat cells engineered with the CD19-CAR (Jurkat-1928z) were used as effector cells. CD19^+^ Raji, CD19^−^ K562, and CD19^+^ K562-CD19 cells were used as target cells. (C) Recombinant retroviruses encoding the inducible ZsGreen1 gene were produced by transient co-transfection methods. To evaluate retroviral vector titer, viral supernatant was directly analyzed in a one-step real-time qPCR reaction. (D) Jurkat and Jurkat-1928z cells engineered with (w/) or without (w/o) inducible ZsGreen1 expression were co-cultured with target cells at E/T = 1. After 24 hr, ZsGreen1 expression was monitored by flow cytometry. (E) Percentage of ZsGreen1-positive cells and (F) mean fluorescence intensity were calculated by flow cytometric analysis. *p < 0.05, ^†^p < 0.01 Data are presented as means ± SEM.

We constructed numerous self-inactivating (SIN) retroviral vectors containing four or six NFAT response elements (NFAT-REs), followed by the minimal IL-2 promoter and a reporter gene (Figure 1A). We also constructed and evaluated other inducible promoters, including the CD28 response element within the IL-2 promoter as well as the Bcl-xL, CD69, and IL-8 promoters, which showed less than optimal responses due to higher basal expression or unresponsiveness following antigen stimulation (data not shown). To test the functionality of NFAT-RE constructs, we used Jurkat and CD19-CAR-expressing Jurkat cells (Jurkat-1928z) as effector cells. We also used K562, CD19-expressing K562, and Raji cells as target cells. CD19-CAR expression was observed in Jurkat-1928z cells, but not in Jurkat cells (Figure 1B). Surface expression of CD19 was observed on CD19-expressing K562 cells and Raji cells. We transduced Jurkat and Jurkat-1928z cells with the SIN-(NFAT)x-ZsGreen1-containing retroviruses (iZsGreen1). To reduce basal expression of transgene background reduction signal (BRS) that is deleted, a hypothetical polyadenylation sequence, “AATAAA,” in antisense orientation from original SV40 early poly(A) was inserted upstream of the inducible promoter. Although there was concern that this modification would affect viral production, high-titer viral supernatants were successfully obtained by transient transfection methods (Figure 1C). The transduction efficiency was estimated by ZsGreen1 expression after stimulation with 12-O-tetradecanoylphorbol-13-acetate (TPA)/ionomycin or stimulation with anti-CD3 and anti-CD28 antibody, which was almost 90% (Figures 1D and 1E). Jurkat-1928z cells transduced with each construct were co-cultured with target cells for 24 hr. Induction of ZsGreen1 expression was observed in cells transduced with each construct by co-culture with CD19-positive target cells. Strikingly, baseline levels of ZsGreen1 expression in cells transduced with the BR-4N construct were reduced compared with cells transduced with other constructs (Figure 1F).

**Figure 2:**
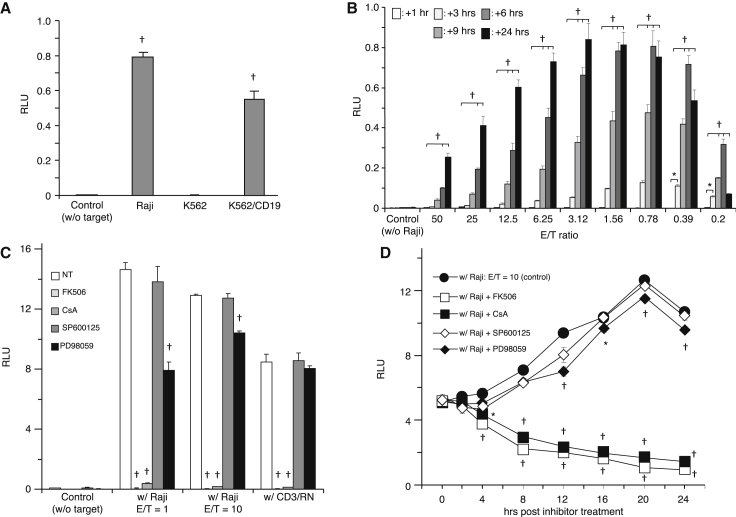
Antigen-Specific Activation of the BR-4N Construct in Jurkat Cells (A) Jurkat-1928z cells engineered with inducible ELuc expression (Jurkat-1928z-iELuc) were co-cultured with target cells at E/T = 1. After 24 hr, ELuc expression was measured by a luciferase assay. (B) Jurkat-1928z-iELuc cells (1 × 10^4^ cells/well) were co-cultured with Raji cells (0.2 × 10^4^–50 × 10^4^ cells/well). Luminescence intensity was measured at the indicated times. Data represent the mean ± SEM of quadruplicates. (C) Jurkat-1928z-iELuc cells were pre-treated with calcineurin inhibitors (FK506 or CsA), a JNK inhibitor (SP600125), or MEK inhibitor (PD98059) for 30 min at 37°C. Then the treated cells were co-cultured with Raji cells at E/T = 1 and 10. OKT-3 and RetroNectin-coated wells were used as positive controls. After 9 hr, luminescence intensity was measured. Data represent the mean ± SEM of quadruplicates. (D) Jurkat-1928z-iELuc cells were co-cultured with Raji cells at E/T = 1 for 8 hr. Then, signaling pathway inhibitors were added. *p < 0.05, ^†^p < 0.01, compared with the control group.

Next, to test the functions of the BR-4N construct quantitatively, we used emerald luciferase (ELuc) as a reporter gene. Induced ELuc expression was observed by co-culture with Raji cells (204-fold) and K562-CD19 cells (142-fold) compared with the control group (without target cells) (Figure 2A). In time course experiments, to monitor ELuc expression regardless of the number of target cells, ELuc expression was induced (Figure 2B). However, most effective induction of ELuc expression was observed at an effector-to-target (E/T) ratio of around 1, and maximum induction of ELuc expression was reached within 9 hr after co-culture with target cells. Importantly, these activations and subsequent ELuc expression could be blocked by the pharmacological inhibitors FK506 (tacrolimus) and cyclosporin A (CsA), which are widely used to downregulate calcineurin activity (Figure 2C), but not other inhibitors, including SP600125, an inhibitor of Jun N-terminal kinase (JNK), or PD98059, a specific inhibitor of mitogen-activated protein kinase (MAPK)/extracellular signal-regulated kinase (ERK). In addition, ELuc expression that was already induced could be reduced strongly by FK506 (Figure 2D). These results indicate that FK506 is a much more potent inhibitor to block transgene expression from NFAT-induced promoter.

### *In Vitro* Functional Validation of Inducible Promoters Using Peripheral Blood Mononuclear Cells

**Figure 3:**
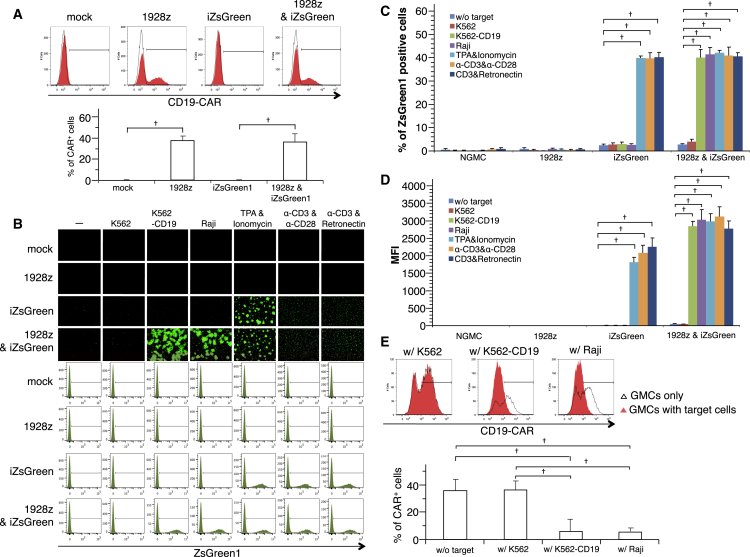
Engineered PBMCs with CAR-Induced ZsGreen1 or ELuc (A) PBMCs were engineered with the CD19-CAR with or without iZsGreen1. CAR expression was measured by flow cytometric analysis. (B) NGMCs or GMCs were co-cultured with target cells. ZsGreen1 expression was measured by flow cytometric analysis. (C) Percentage of ZsGreen1-positive cells and (D) mean fluorescence intensity were calculated by flow cytometric analysis. (E) After co-culture with target cells, CAR expression in GMCs was measured by flow cytometric analysis.

To test the functionality of the BR-4N construct in T cells derived from a healthy donor, we co-transduced T cells with retroviral vectors encoding 1928z or iZsGreen1 genes. The efficiency of CAR expression in CD3-positive T cells was similar regardless of co-transduction with or without iReporter genes and reached about 40% (Figure 3A). Next, CD3-positive T cells were analyzed for their ZsGreen1 expression by flow cytometry. In all iZsGreen1 transduction groups, iZsGreen1 expression was observed by coercive stimulation with TPA and ionomycin, anti-CD3/CD28 antibody, or anti-CD3/RetroNectin (Figures 3B–3D). However, in co-culture experiments, iZsGreen1 expression was only induced strongly in CAR-T/iZsGreen1 cells by co-culture with CD19-positive target cells. Other T cells derived from different donors also showed similar results (data not shown). To ensure that the induced-ZsGreen1 expression was caused by activation signals from the CAR, we examined ZsGreen1 expression in CAR-positive and -negative cells by flow cytometry. However, CAR expression on the cell surface was dramatically reduced after co-culture with CD19-positive target cells (Figure 3E).

**Figure 4:**
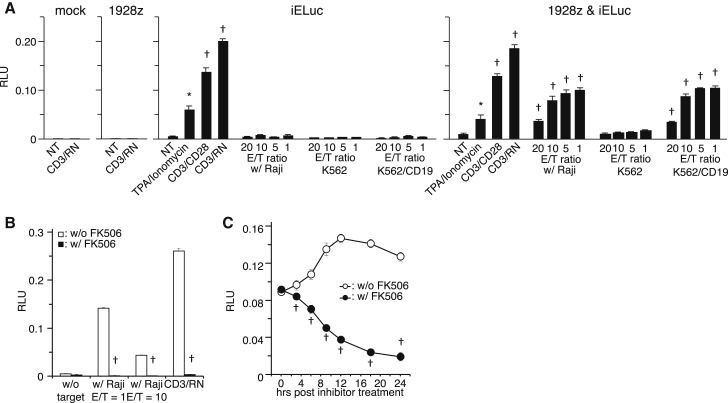
Induction of ZsGreen1 Expression by Co-culture with CD19-Positive Target Cells (A) Engineered PBMCs were co-cultured at increasing numbers (1 × 10^4^–20 × 10^4^ cells/well) with Raji cells (1 × 10^4^ cells/well). After 9 hr, luminescence intensity was measured at the indicated times. Data represent the mean ± SEM of quadruplicates. (B) Engineered PBMCs were pre-treated with FK506 and then co-cultured with Raji cells at E/T = 1 or 10. OKT-3 with RetroNectin-coated wells were used as the positive control. After 9 hr, luminescence intensity was measured. Data represent the mean ± SEM of quadruplicates. (C) Engineered PBMCs were co-cultured with Raji cells at E/T = 1 for 8 hr, and then signaling pathway inhibitors were added. After a further 8 hr of incubation, luminescence intensity was measured. Data represent the mean ± SEM of quadruplicates. *p < 0.05, ^†^p < 0.01, compared with the NT group or w/o the FK506 group.

**Figure 5:**
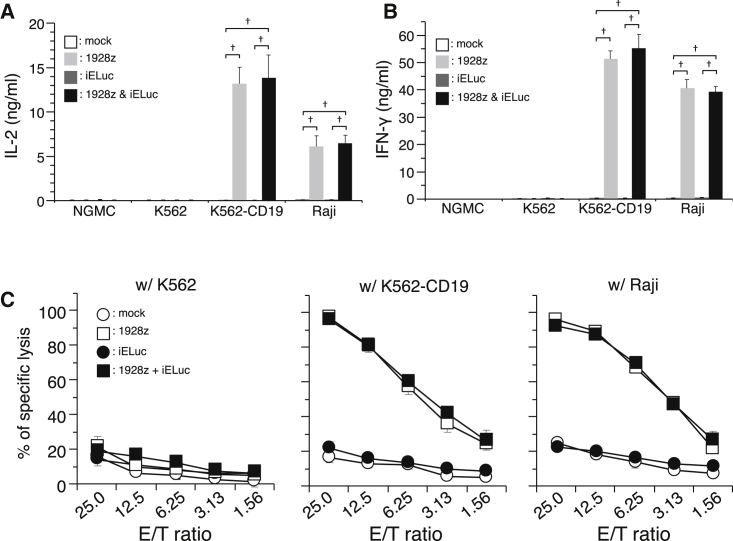
Antigen-Specific Cytokine Release and Cell Lysis Activity of Engineered PBMCs (A) Antigen-specific IL-2 and (B) IFN-γ production was measured by ELISA. Target cells and effector cells were co-cultured at E/T = 1. After 48 hr, supernatants were collected. *p < 0.05. (C) Cell lysis activity of CAR-engineered PBMCs with or without iELuc was assessed by a Calcein AM release-based cytotoxic cell assay. Data are the mean ± SE of triplicate wells.

Next, we substituted the ZsGreen1 gene with the ELuc gene and performed functional validation quantitatively. In all inducible ELuc (iELuc) transduction-only groups, iELuc expression was observed by coercive stimulation with TPA and ionomycin, CD3/CD28, or CD3/RetroNectin (Figure 4A). However, similar to co-culture experiments using iZsGreen1, iELuc expression was strongly induced in CAR-T/iELuc cells by co-culture with CD19-positive target cells. These activations and subsequent reporter gene expression could be blocked by pre-treatment with FK506 (Figure 4B). Importantly, reporter gene expression that was already induced could also be reduced by treatment with FK506 (Figure 4C). Furthermore, after antigen stimulation, CAR-T/iELuc cells produced approximately the same amounts of IL-2 and INF-γ as T cells expressing the CAR only (Figures 5A and 5B). The cells also showed redirected cytolysis toward CD19-positive, but not CD19-negative, tumor cells (Figure 5C).

### *In Vivo* Functional Validation of Inducible Promoters Using Peripheral Blood Mononuclear Cells

**Figure 6:**
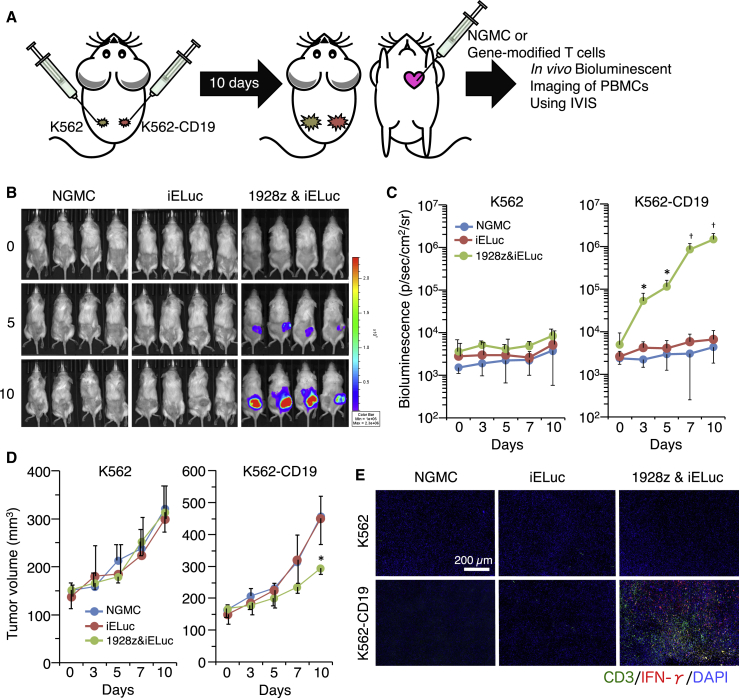
Induction of ELuc Expression at CD19^+^ Tumor Sites (A) Tumor cells were subcutaneously injected into the left (K562) and right (K562-CD19) flanks of NOG mice. At 10 days after tumor cell injection, NGMC, PBMC-iELuc, or PBMC-1928z-iELuc cells were injected into the cardiac chamber. (B) Bioluminescence from PBMCs was periodically measured by *in vivo* imaging. Left, mice injected with NGMC (n = 10). Middle, mice injected with PBMC-iELuc (n = 10). Right, mice injected with PBMC-1928z-iELuc cells (n = 10). (C) Luminescence intensity at tumor sites was measured by Living Image software. *p < 0.05, ^†^p < 0.01, compared with K562 sites. (D) Tumor volumes were calculated by the following formula: tumor volume [mm^3^] = (length [mm]) × (width [mm])^2^ × 0.5 (each groups; n = 10). *p < 0.05, ^†^p < 0.01, compared with the NGMC group. (E) At 5 days after PBMC injection, tumor tissues were collected and subjected to dual immunofluorescence staining for CD3 and IFN-γ.

We assessed the functions of the inducible promoter and target-specific activation of the CAR *in vivo*. NOD/Shi-scid, IL-2Rγnull (NOG) mice were subcutaneously injected with K562 cells (left flank) and K562-CD19 cells (right flank) (Figure 6A). At 10 days after tumor cell injection, gene-modified T cells were systemically infused through the left ventricle, and then luciferase expression was traced over time using an *in vivo* imaging system (IVIS). iELuc expression was strongly induced at CD19-positive tumor sites in the group of administered 1928z/iELuc cells (Figures 6B and 6C). We also assessed the anti-tumor activity of infused T cells. Although the tumors had similar volumes before T cell administration (day 0), significant shrinkage of K562-CD19 tumors was only observed by robust accumulation of 1928z/iELuc cells at day 10 (Figure 6D). At 5 days after T cell administration, we collected tumor tissues from mice and assessed the accumulation and activation of infused T cells. Some T cells were detected at tumor sites in mice that received non-gene-modified cells (NGMCs) or iELuc T cells (Figure 6E). In mice that received 1928z/iELuc cells, a few T cells were detected at K562 tumor sites, whereas numerous IFN-γ-expressing CD3-positive T cells were observed at K562-CD19 tumor sites.

## Discussion

In this study, we describe the molecular function of an inducible promoter driven by activation signals from a CAR. This promoter was selectively driven by activation signals from the CAR. Furthermore, transduction with inducible cassettes, including the inducible promoter and reporter genes, did not affect the original effector activities, including IL-2 and IFN-γ production and antitumor activity of CAR-redirected cytotoxic T lymphocytes.

To generate SIN retroviral constructs, basic cassettes were inserted into a pQCXIX retroviral vector between BglII and *Xho*I restriction sites, where it would be located in the opposite direction of viral gene transcription. Additionally, we inserted two modified SV40 early poly(A) sequences as a BRS upstream of the NFAT-Res, and background expression of the reporter genes was dramatically reduced by insertion of the BRS. Remarkably, the BRS did not affect the production of SIN retroviral vectors or the induced fluorescence intensity of ZsGeen1. When we added two transgenes including the CAR driven by the EF-1α promoter and inducible cassettes in a single vector genome, although we employed many strategies for improvement, it did not work. While we are still very interested in adding a CAR and inducible cassettes in a single vector genome, because of the current circumstances, we transduced T cells with two separate retroviral vectors in this study. We could not determine the cause of the failure, but we considered that interference between the two promoters affects the expression of transgenes contained in a single construct.^28^, ^29^

Retroviral vectors are most widely used for gene transduction in both experimental and clinical studies. In such studies, it is thought that the therapeutic efficacy is directly linked to the transduction efficiency of target cells. RetroNectin is widely used to enhance transduction of various cell types, including lymphocytes, that are difficult to transduce with retroviral vectors by conventional methods.^30^, ^31^, ^32^ Furthermore, stimulation with anti-CD3 and anti-CD28 antibodies is often adopted as a method to expand T cells, but RetroNectin can substitute for an anti-CD28 antibody.^8^, ^33^, ^34^ Although it is still incompletely understood why RetroNectin supports T cell expansion, our results suggest that a combination of RetroNectin and anti*-*CD3 antibody could be used to activate the NFAT signaling pathway in T cells.

Development of novel classes of therapeutic antibodies assumes increasing importance in association with competition in the CAR T cell therapy field. Numerous types of novel antibodies against tumors will be developed in the near future. So far, various NFAT inducible reporter systems have been developed, and usefulness of this system in a combination with CAR have been assessed.^22^, ^26^ This system can be applied not only to use reinforcement of therapeutic efficacy, but also to confirm the antigen specificity of a novel CAR. To use these antibodies in CAR technology, the generation of an scFV, in which the variable heavy (VH) and variable light (VL) domains are joined by a polypeptide linker sequence, is imperative. Generally, they are powerful tools in research and clinical settings owing to better pharmacokinetic properties compared with the parent monoclonal antibodies. Although they offer several advantages, scFV fragments suffer from low binding affinities and rapid clearance from circulation, which limit their therapeutic potential. In CAR strategies, an scFV targeting differentiation antigens can be expected to also recognize non-malignant cells that express the same antigens, resulting in adverse effects.^35^, ^36^ On-target but off-tumor toxicities can be immediately life threatening.^37^, ^38^ It is thought that the fatal toxicity was a result of the high potency of the CAR construct that contained CD28 and 4-1BB, and the use of prior non-myeloablative chemotherapy that further enhanced the treatment effect. Therefore, regardless of the properties of the original antibodies, newly clarifying the avidity and antigen-specific response of a CAR provides a very significant scientific basis for safety and efficacy.

In conclusion, our results indicate that the inducible promoter including four NFAT-REs with a BRS is an appropriate and effective construct that induces exogenous gene expression by activation signals from a CAR. In this study, we almost exclusively used reporter genes as exogenous genes and visualized and quantified the activation status of gene-modified peripheral blood mononuclear cells (PBMCs). The construct is certainly capable of carrying anti-cancer genes such as the IL-12 or IL-18 gene.^22^, ^23^, ^24^, ^25^, ^26^, ^27^ Such an approach may lead to therapeutic strategies that are safe and effective, because exogenous gene expression is confined to the tumor locality.

## Materials and Methods

### Construction of Retroviral Vectors

We prepared two basic inducible promoters that were arranged in the order of multiple (four or six) NFAT-REs in the IL-2 promoter (−278 to −249 nt), a minimal IL-2 promoter (−63 to +51 nt), the reporter gene encoding ZsGreen1 (Clontech Laboratories, Mountain View, CA) or ELuc (Toyobo, Osaka, Japan), and a BGH poly(A) signal sequence. Additionally, we inserted two modified SV40 early poly(A) sequences as a BRS upstream of the NFAT-binding sites. Details of NFAT-REs and modified SV40 early poly(A) sequences are included in the Supplemental Information. To generate SIN retroviral constructs, the inducible promoter was inserted into a pQCXIX retroviral vector (Clontech Laboratories) between BglII and *Xho*I restriction sites, where it would be located in the opposite direction of viral gene transcription. To produce SIN retroviral vectors with inducible promoters (RV-iReporter), a plasmid including the inducible promoter was co-transfected with G glycoprotein of the vesicular stomatitis virus (pVSV-G) and gag-pol (pGP) into 293T cells using the calcium phosphate transfection method. We also prepared a CD19-CAR-expressing retroviral vector (RV-CAR). The 1928z sequence from pSFG-1928z was subcloned into pMEI-5. To produce RV-CAR, pMEI-5-1928z was co-transfected with pVSV-G and pGP into 293T cells using the calcium phosphate transfection method. Then, PG13 viral producer cells were established by stable transduction of VSV*-*G pseudotyped RV-CAR.

### Cell Lines

Jurkat E6.1 cells (European Collection of Cell Cultures, Salisbury, UK), K562 (RIKEN BioResource Center, Ibaraki, Japan), and CD19/K562 cells that were generated by transduction of K562 cells with human CD19-expressing retrovirus vectors were grown in RPMI 1640 medium (Life Technologies, Gaithersburg, MD) supplemented with 10% fetal bovine serum (FBS), 100 U/mL penicillin, and 100 μg/mL streptomycin (P/S) (RPMI complete medium). 293T cells (RIKEN BioResource Center) were cultured in DMEM/F-12 medium (Life Technologies) supplemented with 10% FBS and P/S (DMEM/F-12 complete medium). All cultures were maintained in an incubator at 37°C with 5% CO_2_.

### PBMCs

Peripheral blood (30 mL) was obtained from three healthy donors who provided informed consent. PBMCs were separated with lymphoprep (Axis Shield, Oslo, Norway) and washed twice with Cellotion (Zeanoq, Fukushima, Japan). The PBMCs were re-suspended in cryopreservation medium consisting of CP-1 (Kyokutou Seiyaku, Tokyo, Japan), RPMI 1640, and human serum albumin (Albuminar; CSL Behring, Marburg, Germany). PBMCs were frozen and stored in liquid nitrogen until further use. The PBMCs were cultured in GT-T503 (Takara Bio., Shiga, Japan) supplemented with 0.6% autologous plasma, 0.2% Albuminar, 1× Antibiotic-Antimycotic (Life Technologies), and 175 IU/mL IL-2 (Immunase; Shionogi & Co., Tokyo, Japan). PBMCs were used to generate gene-modified PBMCs (GMCs) and non-gene-modified PBMCs (NGMCs).

### Propagation of GMCs

For retroviral transduction, PBMCs were stimulated with immobilized anti-CD3 antibody OKT-3 (eBiosciences, San Diego, CA) and RetroNectin (Takara Bio) for 4 days. Then, we prepared three groups of retroviral mixtures including RV-CAR only, RV-iReporter only, and a 1:1 mixture of RV-CAR and RV-iReporter. These mixtures were applied to vector-preloaded RetroNectin-coated 24-well plates and centrifuged at 2,000 × *g* for 2 hr at 32°C. Then, pre-stimulated PBMCs were added to the preloaded plates and centrifuged at 1,000 × *g* for 10 min at 32°C. Cells were cultured at 37°C for 5 hr and then transferred to T-25 culture flasks (BD Falcon).

### Flow Cytometry

CAR expression was monitored by flow cytometry using a biotin-conjugated anti-mIgG1 antibody (Jackson ImmunoResearch Laboratories, West Grove, PA) and phycoerythrin (PE)-conjugated streptavidin (Beckman Coulter, Marseille, France) with an LSRFORTESSA (BD Bioscience, San Diego, CA).

### ELISA

Supernatants from co-cultures of NGMCs or GMCs with target cells at a 1:1 ratio were harvested after 48 hr of incubation. Production of IL-2 and IFN-γ were measured by ELISA kits (Thermo Fisher Scientific, Waltham, MA).

### Cytotoxic T Lymphocyte Assay

To monitor cytolytic activity, increasing numbers of CAR-expressing PBMCs were co-cultured with tumor cells for 6 hr in 96-well plates. In brief, tumor cells as target cells were resuspended in complete medium at a final concentration of 1 × 10^6^/mL and incubated with 15 μM of calcein-AM (Dojindo Lab, Kumamoto, Japan) for 30 min at 37°C. After two washes in complete medium, the cells were adjusted to 1 × 10^6^/mL. The assay was performed in V-bottom 96-well microtiter plates (Corning) with E:T ratios ranging from 25:1 to 1.56:1 in triplicate, and triplicate wells for spontaneous (target cells only in complete medium) and maximum release (target cells only in medium plus 2% Triton X-100). Various numbers of PBMCs as effector and target cells were seeded as follows: for the macroassay (standard), each well contained 1.56 × 10^4^–2.5 × 10^5^ lymphocytes in 100 μL of complete medium and 1 × 10^4^ target cells/50 μL of complete medium. After incubation at 37°C with 5% CO_2_ for 6 hr, 75 μL of each supernatant was harvested and transferred into a flat-bottom 96-well plate. Samples were analyzed using a fluoroscan (excitation filter, 485 nm; band-pass filter, 538 nm; Thermo Fisher Scientific). Data were expressed as arbitrary fluorescent units (FU). Specific lysis was calculated according to the formula ([test release − spontaneous release]/[maximum release − spontaneous release]) × 100.

### *In Vitro* Reporter Assay

Jurkat-1928z-iReporter cells or GMCs were co-cultured with target tumor cells for the times indicated in figure legends. Luciferase activity was measured by the Bright-Glo Luciferase Assay System (Promega, Madison, WI) with the fluoroscan. In the inhibition experiments, Jurkat-1928z-iReporter cells or GMCs were treated with calcineurin inhibitor (100 nM FK506; InvivoGen, San Diego, CA) or 100 nM cyclosporine A (CsA) (Cell Signaling Technology, Danvers, MA), 50 μM c-Jun N-terminal kinase (JNK) inhibitor (SP600125; Cell Signaling Technology), or 50 μM mitogen-activated protein kinase (MEK) inhibitor (PD98059; Cell Signaling Technology).

### *In Vivo* Imaging

NOG mice were purchased from the Central Institute for Experimental Animals (Tokyo, Japan). K562 cells were subcutaneously injected into the left flank of NOG mice, while K562-CD19 cells were subcutaneously injected into the right flank of the same mouse. Ten days after tumor cell injection, GMCs were injected into the cardiac chamber. Optical bioluminescence imaging was performed to periodically trace the cells using an IVIS (Xenogen, Alameda, CA). To detect bioluminescence from GMCs, the reporter substrate D-luciferin (Ieda Chemical, Tokyo, Japan) was injected into the mouse peritoneum (75 mg/kg body weight) for scanning. The luminescent intensity at tumor sites was analyzed using Living Image software (Xenogen).

### Immunohistochemistry

K562 cells were subcutaneously injected into the left flank of NOG mice, while K562-CD19 cells were subcutaneously injected into the right cavity of the same mouse. Ten days after tumor cell injection, GMCs were injected into the cardiac chamber. Five days after GMC injection, mice were sacrificed, and paraffin*-*embedded tissues were prepared. Immunohistochemistry was performed with an anti-CD3 antibody (ab109531; Abcam, Cambridge, MA) and Alexa Fluor 488 chicken anti-rabbit immunoglobulin G (IgG) (Abcam) to detect injected GMCs. We also performed immunohistochemistry with an anti-IFN-γ antibody (AF-285-NA; R&D Systems, Minneapolis, MN) and Alexa Fluor 594 donkey anti-goat IgG (Abcam) to detect activated GMCs. Nuclei were stained with DAPI (SlowFade Goldantifade reagent with DAPI; Thermo Fisher Scientific). Images were obtained with a fluorescence microscope (VS120-L100; Olympus, Tokyo, Japan).

### Statistics

Mean values and SDs were calculated using StatMate (Atms, Tokyo, Japan). Significant differences were assessed by the Student’s t test. p < 0.05 was considered to be statistically significant.

## Author Contributions

Conceptualization, R.U., K. Ozawa; Methodology, R.U., T.T., H.I.; Investigation, R.U., T.T., H.I., K. Ohmine; Writing – Original Draft, R.U., K. Ohmine, K. Ozawa; Writing – Review & Editing, Y.S., M.U., H.M.; Funding Acquisition, R.U., K. Ozawa; Resources, J.M.; Supervision, R.U., J.M., and K. Ozawa.

## Conflicts of Interest

Three of the authors (R.U., K. Ohmine, and K. Ozawa) are supported by Takara Bio, Inc. Three of the authors (T.T., H.I., and J.M.) are employed by Takara Bio, Inc. The other authors declare no competing interests.

## Supplemental Information

Document S1. Supplemental Materials and Methods

Document S2. Article plus Supplemental Information
