# Supplementary material for: Functional Analysis of an Inducible Promoter Driven by Activation Signals from a Chimeric Antigen Receptor
Source: Mol Ther Oncolytics. 2018 Dec 1;12:16–25. doi: 10.1016/j.omto.2018.11.003 (PMC6325072; doi:10.1016/j.omto.2018.11.003)
Supplement: Document S1. Supplemental Materials and Methods [file mmc1.pdf]

OMTO, Volume *12*

Supplemental Information

Functional Analysis of an Inducible Promoter Driven by Activation Signals from a Chimeric Antigen Receptor

Ryosuke Uchibori, Takeshi Teruya, Hiroyuki Ido, Ken Ohmine, Yoshihide Sehara, Masashi Urabe, Hiroaki Mizukami, Junichi Mineno, and Keiya Ozawa

Supplementary information

■NFAT

GGAGGAAAAACTGTTTCATACAGAAGGCGT

■Minimal IL-2 promoter

CAGAATTAACAGTATAAATTGCATCTCTTGTTCAAGAGTTCCCTATCAC
TCT

■Original SV40 early polyA

GATCATAATCAGCCATACCACATTTGTAGAGGTTTTACTTGCTTTAAAA
AACCTCCCACACCTCCCCCTGAACCTGAAACATAAAATGAATGCAATTG
TTGTTGTTAACTTG**TTTATT**GCAGCTTATAATGGTTACAAATAAAGCAA
TAGCATCACAAATTTCACAAATAAAGCATTTTTTTCACTGCATTCTAGTT
GTGGTTTGTCCAAACTCATCAATGTATCTTATCATGTCTG

■Modified polyA (BRS): A hypothetical polyadenylation sequence 'AATAAA' in antisense orientation was eliminated from original poyA.

GATCATAATCAGCCATACCACATTTGTAGAGGTTTTACTTGCTTTAAAA
AACCTCCCACACCTCCCCCTGAACCTGAAACATAAAATGAATGCAATTG
TTGTTGTTAACTTGCAGCTTATAATGGTTACAAATAAAGCAATAGCATC
ACAAATTTCACAAATAAAGCATTTTTTTCACTGCATTCTAGTTGTGGTTT
GTCCAAACTCATCAATGTATCTTATCATGTCTG

■4N

GGAGGAAAAACTGTTTCATACAGAAGGCGTGGAGGAAAAACTGTTTCA
TACAGAAGGCGTGGAGGAAAAACTGTTTCATACAGAAGGCGTGGAGGA
AAAACTGTTTCATACAGAAGGCGTccccgggacattttgacacccccataatatttttcCA
GAATTAACAGTATAAATTGCATCTCTTGTTCAAGAGTTCCCTATCACTC
Tctttaatcactactcacagtaacctcaactcctgaattcc[gene of interest]

■BR-4N

GATCATAATCAGCCATACCACATTTGTAGAGGTTTTACTTGCTTTAAAA
AACCTCCCACACCTCCCCCTGAACCTGAAACATAAAATGAATGCAATTG

TTGTTGTTAACTTGGCAGCTTATAATGGTTACAAATAAAGCAATAGCAT
CACAAATTTCACAAATAAAGCATTTTTTTCACTGCATTCTAGTTGTGGTT
TGTCCAAACTCATCAATGTATCTTATCATGTCTGGATCATAATCAGCCA
TACCACATTTGTAGAGGTTTTACTTGCTTTAAAAAACCTCCCACACCTC
CCCCTGAACCTGAAACATAAAATGAATGCAATTGTTGTTGTTAACTTGG
CAGCTTATAATGGTTACAAATAAAGCAATAGCATCACAAATTTCACAAA
TAAAGCATTTTTTTCACTGCATTCTAGTTGTGGTTTGTCCAAACTCATCA
ATGTATCTTATCATGTCTGaccggtcgGGAGGAAAAACTGTTTCATACAGAA
GGCGTGGAGGAAAAACTGTTTCATACAGAAGGCGTGGAGGAAAAACTG
TTTCATACAGAAGGCGTGGAGGAAAAACTGTTTCATACAGAAGGCGTccc
cgggacattttgacacccccataatatttttcCAGAATTAACAGTATAAATTGCATCTCT
TGTTCAAGAGTTCCCTATCACTCTctttaatcactactcacagtaacctcaactcctgaattc
c[gene of interest]

■6N
GGAGGAAAAACTGTTTCATACAGAAGGCGTGGAGGAAAAACTGTTTCA
TACAGAAGGCGTGGAGGAAAAACTGTTTCATACAGAAGGCGTGGAGGA
AAAACTGTTTCATACAGAAGGCGTGGAGGAAAAACTGTTTCATACAGAA
GGCGTGGAGGAAAAACTGTTTCATACAGAAGGCGTccccgggacattttgacacc
cccataatatttttcCAGAATTAACAGTATAAATTGCATCTCTTGTTCAAGAGTT
CCCTATCACTCTctttaatcactactcacagtaacctcaactcctgaattcc[gene of interest]

■BR-6N
GATCATAATCAGCCATACCACATTTGTAGAGGTTTTACTTGCTTTAAAA
AACCTCCCACACCTCCCCCTGAACCTGAAACATAAAATGAATGCAATTG
TTGTTGTTAACTTGGCAGCTTATAATGGTTACAAATAAAGCAATAGCAT
CACAAATTTCACAAATAAAGCATTTTTTTCACTGCATTCTAGTTGTGGTT
TGTCCAAACTCATCAATGTATCTTATCATGTCTGGATCATAATCAGCCA
TACCACATTTGTAGAGGTTTTACTTGCTTTAAAAAACCTCCCACACCTC
CCCCTGAACCTGAAACATAAAATGAATGCAATTGTTGTTGTTAACTTGG
CAGCTTATAATGGTTACAAATAAAGCAATAGCATCACAAATTTCACAAA
TAAAGCATTTTTTTCACTGCATTCTAGTTGTGGTTTGTCCAAACTCATCA
ATGTATCTTATCATGTCTGaccggtcg

GGAGGAAAAACTGTTTCATACAGAAGGCGTGGAGGAAAAACTGTTTCA
TACAGAAGGCGTGGAGGAAAAACTGTTTCATACAGAAGGCGTGGAGGA
AAAACTGTTTCATACAGAAGGCGTGGAGGAAAAACTGTTTCATACAGAA
GGCGTGGAGGAAAAACTGTTTCATACAGAAGGCGTccccgggacattttgacacc
cccataatatttttcCAGAATTAACAGTATAAATTGCATCTCTTGTTCAAGAGTT
CCCTATCACTCTctttaatcactactcacagtaacctcaactcctgaattcc[gene of interest]
